# Supplementary material for: Large carbon sink potential of secondary forests in the Brazilian Amazon to mitigate climate change
Source: Nat Commun. 2021 Mar 19;12:1785. doi: 10.1038/s41467-021-22050-1 (PMC7979697; doi:10.1038/s41467-021-22050-1)
Supplement: Supplementary file 1 — Supplementary Information [file 41467_2021_22050_MOESM1_ESM.pdf]

Supplementary Information for:

Large carbon sink potential of Secondary Forests in the Brazilian Amazon to mitigate climate change

Authors

Viola H. A. Heinrich[1*], Ricardo Dalagnol[2], Henrique L. G. Cassol[2], Thais M. Rosan[3], Catherine Torres de Almeida[2], Celso H. L. Silva Junior[2], Wesley A. Campanharo[2], Joanna I. House[1,4], Stephen Sitch[3], Tristram C. Hales[5], Marcos Adami[6], Liana O. Anderson[7], Luiz E. O. C. Aragão[2,3]

Affiliations and Addresses

[1]School of Geographical Sciences, University of Bristol, Bristol UK.

[2]Remote Sensing Division, National Institute for Space Research (INPE), São José dos Campos, Brazil.

[3]College of Life and Environmental Sciences, University of Exeter, Exeter, UK.

[4]Cabot institute, University of Bristol, Bristol, UK.

[5]School of Earth and Ocean Sciences, Cardiff University, Cardiff, UK.

[6]Amazon Regional Center, National Institute for Space Research (INPE), Belém, Brazil.

[7]National Center for Monitoring and Early Warning of Natural Disaster, São José dos Campos, Brazil.

Corresponding author Contact:

*Email: viola.heinrich@bristol.ac.uk

This Supplementary Information includes:

- Supplementary Notes (p2 – p5)
- Supplementary Figures (p6 – p12)
- Supplementary Tables (p12 – p16)
- Supplementary References (p16 – p20)

Supplementary Notes

Supplementary Note 1: Method for identifying Secondary Forests

The method for identifying secondary forests used in this research has only been used twice prior to this study and has never been applied in further analysis[1,2]. It was therefore important to consider how this method compared to areas classified as secondary forest from a widely used land cover product in Brazil, TerraClass[3]. We did not consider using TerraClass as the primary land-use land-cover dataset in this research since the maps are only available from 2000 and at 4-year intervals until 2014, not providing us with the annual temporal resolution needed in this research. It was therefore used purely for comparative and validation purposes. We did this by visual inspection on the biome scale (Supplementary Figure 1), the local scale (Supplementary Figure 2) and through statistical analysis (Supplementary Figure 3).

The majority of secondary forests identified were in eastern Amazonia (Supplementary Figure 1), this is in line with previous research in this field[3–5]. The total area of secondary forest in 2017 was 14 Mha. Again, this value is very similar to recent research which applied the same approach (12 Mha)[1]. Results show the similarity of secondary forest identified using our approach and that by TerraClass (Supplementary Figures 1 – 3). However, some areas were identified differently such as areas of perennial agriculture (Oil palm plantations) and Silviculture (Supplementary Figure 2), which is in part a consequence of the automated nature of the MapBiomas classification system. The statistical analysis shows that there is a strong linear relationship between the two products (n = 5,868 grid squares of 0.25°; Pearson's $r = 0.7$; Supplementary Figure 3). This is a promising result suggesting that, given the automated nature of MapBiomas, it has the potential for wider applications and in other regions[2] and countries (see "MapBiomas Amazonia": http://amazonia.mapbiomas.org/ and "MapBiomas Indonesia": http://nusantara.earth/).

Supplementary Note 2: Secondary forest regrowth estimates using remote sensing data

We initially used two remote sensing products to estimate the regrowth of secondary forest with age and compared their relative applicability for this study. The first Aboveground Biomass (AGB) product we evaluated was based on the methodology from Baccini et al.[6], which estimates the AGB at 30m resolution for 2000[7] (hereon- AGB-Baccini). This product has been widely used for AGB studies across the tropics and cited ~1300 times. The ages of secondary forest were constructed for the year 2000, meaning the maximum age was 15 years (if regrowing since 1986). Given that the AGB-Baccini product has the same resolution as the MapBiomas product, the AGB pixels corresponding to the overlying secondary forest were directly extracted and evaluated by age. The second product we evaluated was the European Space Agency Climate Change Initiative (ESA-CCI) AGB product (see *Methods* of main paper).

For computing purposes and to begin to capture spatial variability in regrowth, Amazonia was split up into six sectors (Supplementary Figure 5 – 6: inlet). Importantly, analysis of both products shows that the AGB across the secondary forest areas is generally lower than those of the old-growth forest values (Supplementary Figures 5 – 6). This finding is in line with the natural pattern of regrowth and is a promising result for distinguishing secondary forest AGB from old-growth forest AGB using remote sensing data. Further analysis using the AGB-Baccini dataset showed that the AGB of secondary forests, regardless of age, was very high with a mean AGB value of up to 200Mg ha^{-1} for a 1 year old secondary forest (Supplementary Figure 5), considerably higher than other commonly used estimates of AGB in the Brazilian Amazon, which is about 20 Mg ha^{-1} in secondary forest less than 5 years old and 150 to 200 Mg ha^{-1} in forests with an age of 15 - 20 years [8,9]. This observation was true for all sectors into which Amazonia was divided (Supplementary Figure 5). While previous research has shown that the AGB-Baccini dataset generally overestimates AGB compared to other datasets[10], there has been no study of the impact of this on the estimation of secondary forest regrowth. Our results

suggest that caution should be taken when using the AGB-Baccini dataset for analysis of secondary forest. Going forward in the research we decided that the ESA-CCI AGB dataset was more appropriate to use for our analysis, which showed the increase in AGB with age in all sectors, and showed lower AGB values compared to AGB-Baccini, closer to what is expected for the respective ages[8,9]. In addition, it enabled us to use the entire secondary forest age range created from MapBiomas (1 to 32 years).

Supplementary Note 3: Remote sensing product uncertainties

We briefly analysed the accuracy of the MapBiomas dataset for Amazonia and found an overall accuracy of 95.8% for the land cover classification. Reductions to the overall accuracy were due to allocation disagreements (2.3%) and area disagreements (1.9%)[11].

Here we provide more detail on the ESA-CCI AGB product origin and the associated uncertainties of this new product given its recent development. The ESA-CCI AGB map for 2017 shows AGB defined as the mass, or dry-oven weight, of the woody parts (including the stem, bark, branches, and twigs) of all living trees. This definition excludes the stump and roots[12]. The ESA-CCI AGB product relies on initial Synthetic Aperture Radar (SAR) backscatter data from the C-band Sentinel-1 and the L-band Advanced Land Observing Satellite (ALOS-2) Phased Array L-band SAR (PALSAR-2). The datasets from both remote sensing products were converted using an algorithm that relates specific forest backscatter components and properties to Growing Stock Volume (GSV) using key auxiliary datasets of canopy density, microwave transmissivity and land-cover, among others. GSV is the volume of all living trees within an area (m^3 ha^{-1}). These maps were then merged and converted to AGB using a conversion factor, which accounts for wood density to convert volume to mass, and an expansion factor, which considers the proportion of stem biomass to total biomass[12]. The product was validated against 49,796 forest plots across the world[12]. Most of the forest plots were from: Western Australia, China, Western Europe, Alaska, Central Africa and Amazonia. Two maps are then produced, the AGB estimates and the standard deviation, both of which are expressed in Mg ha^{-1}. Variations in the AGB product is a result of two things. The first is that the products used to estimate AGB are only indirectly related to AGB and therefore introduce uncertainties in the assumptions made to produce the AGB maps. Secondly, uncertainties may be introduced due to measurement errors from the original remote sensing products. These kinds of errors also apply to the other satellite products used in this study. There are also issues related to the under- and over-estimation in dense tropical forests and low biomass forests respectively. In dense tropical forests, the L-band signal saturates at high biomass values, whilst in low biomass regions the model framework and parametrization lead to uncertainties in the AGB estimates[12].

Additionally, there are further technical limitations relating to the temporal and spatial resolution of remote sensing datasets used in this study which add further uncertainty to the estimates and regrowth curves. We briefly assessed the standard deviation of the original ESA-CCI AGB product in old-growth forests and secondary forest of Amazonia. For old-growth and secondary forest, we find that the average standard deviation is approximately 68% and 78%, respectively. These values show that the spread around the AGB is considerably large due to the reasons explained above as well as varying forest and land-use land-cover dynamics. They also highlight the need to compare AGB values with other models and field data plots, as we have done in this study. By comparing our approach with other method provides additional validation to the method developed in this study.

Supplementary Note 4: Validation of Regrowth models

We used field data of secondary forest, their ages and Aboveground Carbon (AGC), collected across Amazonia, to compare and validate our spatially explicit models (Supplementary Table 10; Supplementary Figure 10)[8]. We compare secondary forests identified in our study within a 3km radius of the field data. In both datasets we removed secondary forests which had experienced repeated deforestations or burning and only considered non-disturbed secondary forests. We grouped the two datasets into age ranges (1 to 8, 8 to 16, 16 to 24, 24 to 32 years) and compared the spread of the AGC

data. We also calculated whether the median AGC for the two datasets was statistically significantly different by applying the non-parametric Wilcoxon test, with a p-value <0.01 indicating that the difference between the medians is statistically significant.

For all regions, the AGC estimates evaluated in this study and the field data were statistically similar (Supplementary Figure 10). However, by visual inspection a noticable difference between the AGC values in the older secondary forests in the North-Eastern region can be seen (Supplementary Figure 10b), where the interquartile ranges of the two datasets do not overlap. This difference may be linked to the fact that there are generally fewer field plots in this region and those that do exist are clustered around one location. Additionally, as explained above, L-band signal saturations at high biomass (carbon) values, generally corresponding to areas of older secondary forest, means the remote sensing data cannot reach values as high as field data estimates. There may also be issues related to the sample size of the field data, whereby a small sample area of secondary forest with a high biomass results in an amplified signal of AGC when the values are converted to a density (Mg C ha^{-1}).

We also compared our models to secondary forest regrowth models used in previous research and in the Brazilian Greenhouse gas Inventories[5,13]. The first is the simple linear model developed by Alves et al., (1997), which was adapted to be used in the Brazilian Greenhouse Gas (GHG) inventory[13,14]:

$$Y(t) = 3.94 \times t \tag{2}$$

The second growth model, was the hyperbolic carbon model used by Wang et al. (2020):

$$Y(t) = (A \times t)/(\alpha_{50} + t) \tag{3}$$

where Y refers to the AGC at age t (in Mg C ha^{-1}); carbon density $A = 170.6$ Mg C ha^{-1} and α_{50} refers to the age at which approximately half of the maximum carbon sequestration is reached (35 years)[5]. As in Equation (1) of the main paper, t refers to the age of secondary forest.

Our estimates in regions of no disturbance in North- and South-Western Amazonia visually agree with the models used by Wang et al. (2020) and the Brazilian GHG inventory, however these similarities are not always statistically similar (Supplementary Figure 11; Supplementary Table 11). In eastern parts and regions of disturbance, the regrowth rates of the other models are up to 3 MgC ha^{-1} yr^{-1} higher compared to our models (Supplementary Figure 11). This divergence may in part be linked to the models in our study being driven by a set value of old-growth forest AGC (asymptote) obtained from the ESA-CCI product for the region and considering secondary forest disturbance (burning). Additionally, the model used by the GHG inventory uses a value that is specifically derived from data in regions of secondary forest with little prior land-use[13]. In Western and Central regions, our regrowth models under no disturbance conditions are very similar to the model used by Wang et al. (2020), with differences approximately ±1 MgC ha^{-1} yr^{-1} (Supplementary Figure 11). However, our modelled regrowth under disturbance (burning) is considerably lower (Supplementary Figure 11). This highlights the potential importance of being able to disentangle the drivers influencing regrowth in different regions.

Supplementary Note 5: Conditional Random Forest model and variable correlation

To determine which "random forest" model to implement when analysing the variable importance (see *Methods* section of main paper) it was important to consider the input variables and the degree of their correlation between the variables themselves (spatial autocorrelation) and between one another (spatial co-variation). Given that we applied a cluster analysis to create regions of Amazonia based on similarities of the climate variables (shortwave radiation, annual average precipitation, and Maximum Cumulative Water Deficit – MCWD) we anticipated the variables to be spatially auto-correlated. This was simply the nature of the clustering approach.

We also considered the correlation between the variables that overlay a given secondary forest polygon in the sample data (see methods of main paper) by applying the Spearman's rank statistical analysis from a random sample of data points equating to 2% of the secondary forest polygons (Supplementary Figure 13). The results showed that some variables were highly correlated, most noticeably precipitation and MCWD, which have a strong and highly significant positive relationship (up to 0.84; $p<0.001$). The relationship is positive due to the sign of the MCWD (negative), so as precipitation increases, MCWD becomes less negative. This was to be expected given that the former is used to produce the MCWD index. We expected SW radiation and precipitation to be more directly negatively correlated, assuming the lower cloud cover (higher SW radiation) also resulted in less precipitation. However, we found a relatively weak (-0.07 to -0.36) but highly significant correlation ($p<0.001$) across Amazonia (Supplementary Figure 13a – d). We believe this may be linked to the fact that we considered the annual average precipitation and SW radiation and did not consider seasonal variability of these variables, where we would expect a more direct (negative) correlation, especially in the wet season.

We observe a relatively strong, significant, negative correlation between precipitation and Soil Cation Concentration (SCC) (-0.19 to -0.54) as well as MCWD and SCC (-0.07 to -0.5) (Supplementary Figure 13). This correlation may be linked to high precipitation resulting in greater leaching of soil nutrients, especially close to the Andes where much of the soil nutrients originate from[15,16]. Eroded sediments can travel long distances into the Amazon biome and be deposited far from their origin. There are numerous other factors that could cause this relationship and may therefore mean that this correlation is not always causational. For example, regions that are generally dry and have a high SCC, such as the South West (Supplementary Figure 7), are subject to high sediment depositions during La Niña years. In these years, heavy precipitation events in the Bolivian Andes results in soil erosion, and the downstream deposition of nutrient rich sediments[17]. However, detailed assessment of the individual correlations and causations of each of the variables is not within the scope of this study.

Given that some of the variables had a strong and significant relationship we opted to use the conditional random forest model (available in the programme R within the 'party' and 'caret' packages called 'cforest')[18–20]. This model was applicable in this research as it provides more accurate assessments if (i) the variables are of different types (continuous and categorical) and if (ii) the variables are highly correlated. Given that the variables in this study were both categorical and continuous and/or correlated up to some degree, this specific model was considered the most suitable. Research which developed the cforest model highlighted that there are two possible reasons for more traditional random forests models to have a bias towards correlated and continuous variables or categorical variables with numerous categories[19]. Firstly, they found that during the tree building process, there was a preference for the selection of correlated variables as well as variables with a high number of categories, which have a higher potential for a greater number of 'cutpoints'[18–20]. They also found that during the assessment of variable importance, importance of correlated variables were overestimated in unconditional permutations[18].

Cforest addresses these problems by developing a statistical framework that decouples correlated variable importance better than traditional random forest models. It uses unbiased decision trees and a resampling scheme by default. During the assessment of variable importance, it uses the so-called conditional permutation scheme, whereby the impact of each individual predictor variable on the response variable is accounted for irrespective of any correlation with another variable. In each permutation, the relationship between each individual predictor variable is tested and only the variable with the lowest P-value is carried forward to determine the importance at the end of the permutations[21].

This made the cforest model a suitable candidate in this research, however it is important to stress that the conditional permutation is a computationally expensive task, it was therefore important to find the balance between computation speed and accuracy of results (see *Methods* section in main paper).

Supplementary Figures

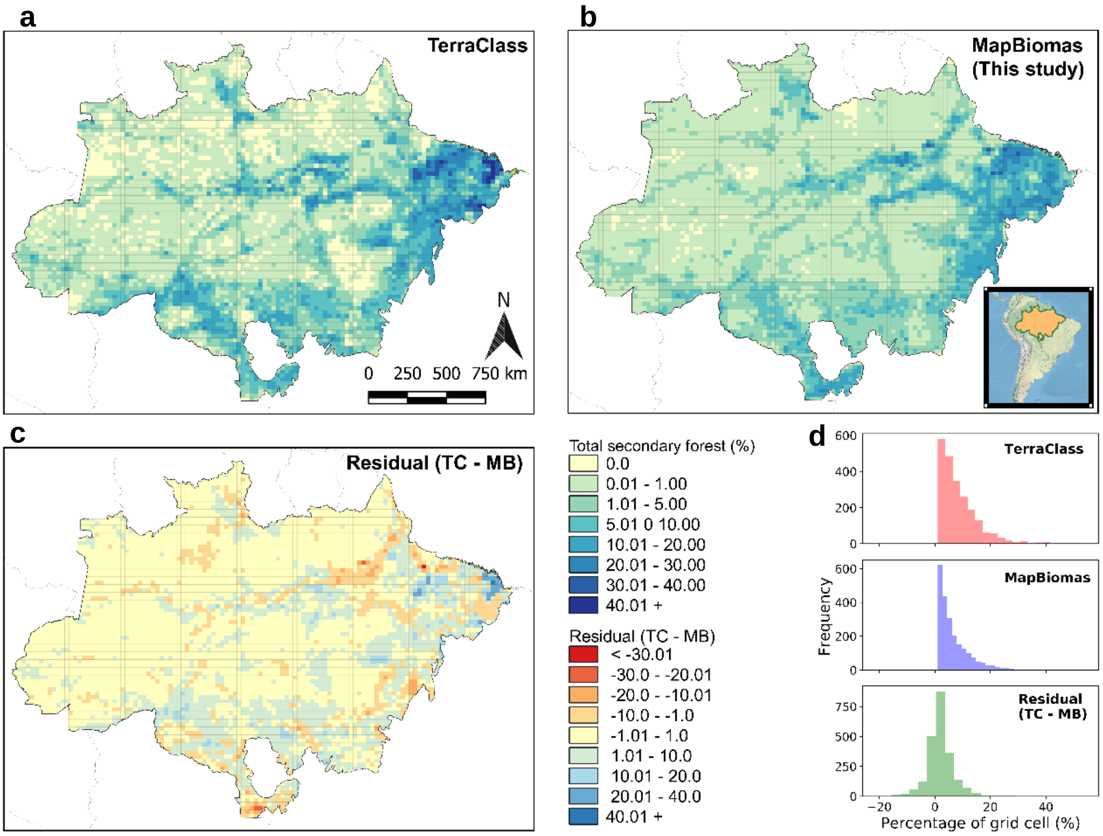

Supplementary Figure 1 | Secondary forest identification in Amazonia, Brazil. Secondary forests in 2014 as identified by (**a**) TerraClass, (**b**) MapBiomas (this study), and (**c**) the residual of the two dataset (TerraClass (TC) minus MapBiomas (MB)). The values shown are all in percentage of secondary forest occupied within a 0.25° grid (seen in figure) and (**d**) shows the corresponding frequency distribution of secondary forest occupied in each grid cell for (**a-c**).

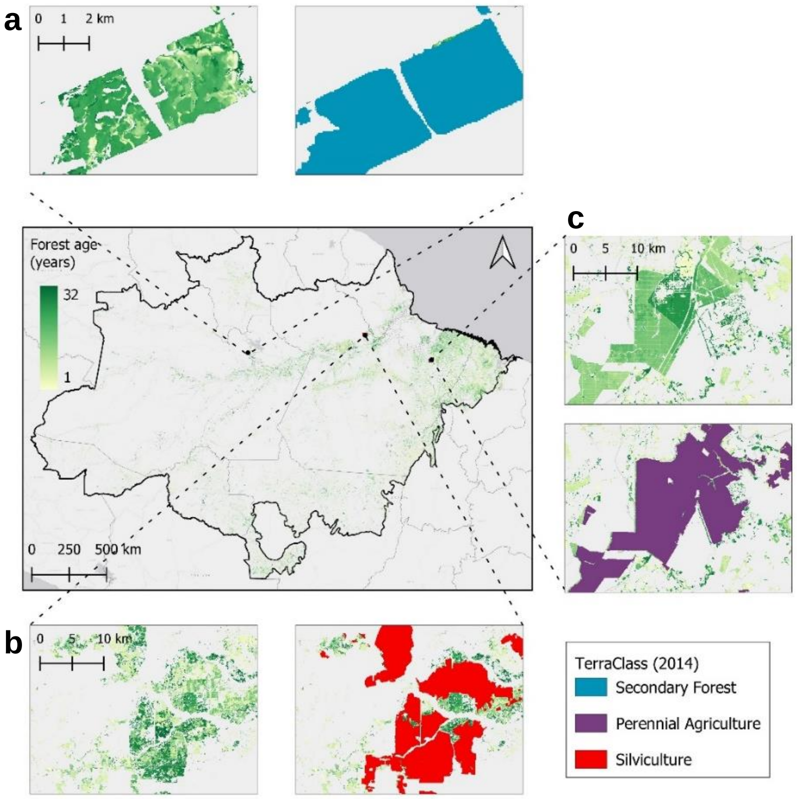

Supplementary Figure 2 | Local scale analysis of Secondary Forests. Examples of regions identified as Secondary Forest using MapBiomas v3.1 land cover data and methods described in main Methods section within the Brazilian Amazon Biome (main map) and how this identification compares with TerraClass 2014. A region (**a**) identified as secondary forest by both products, (**b**) a region identified as Silviculture by TerraClass 2014, and (**c**) a region identified as Perennial Agriculture (Oil Palm plantation) by TerraClass 2014.

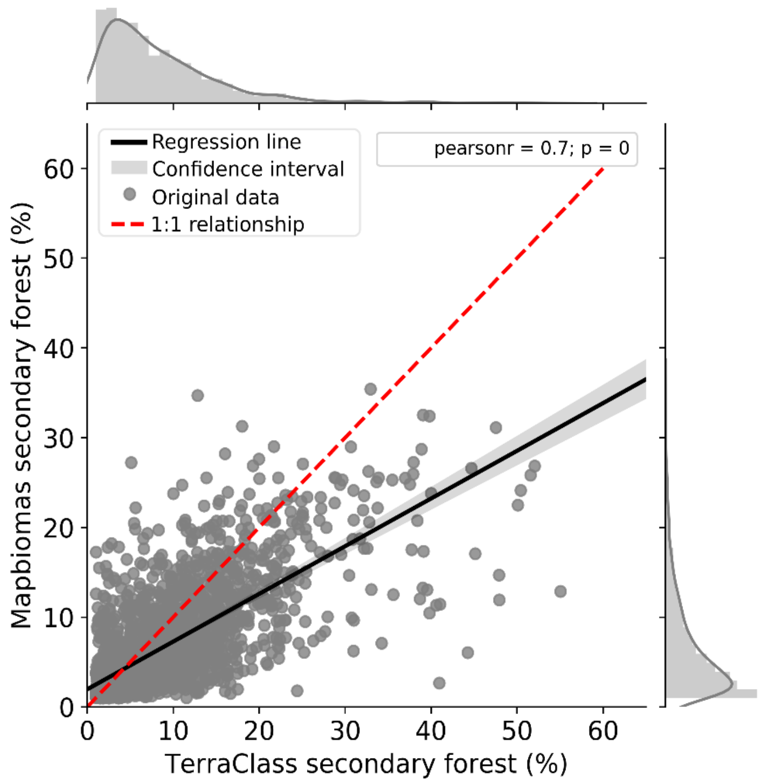

Supplementary Figure 3 | Correlation of percentage of secondary forest in the year 2014. The scatter graph shows the percentages for each (5,868 total) 0.25° grid boxes (seen in Supplementary Figure 1), comparing the widely used land cover product (TerraClass) and the secondary forest as identified in this research using MapBiomasV3.1. The black line shows the regression calculated using the individual points and the associated 95% confidence intervals (grey shading). The red dotted line shows the perfect 1:1 relationship. Additionally, a schematic histogram is shown, highlighting the spread in the percentage of secondary forest.

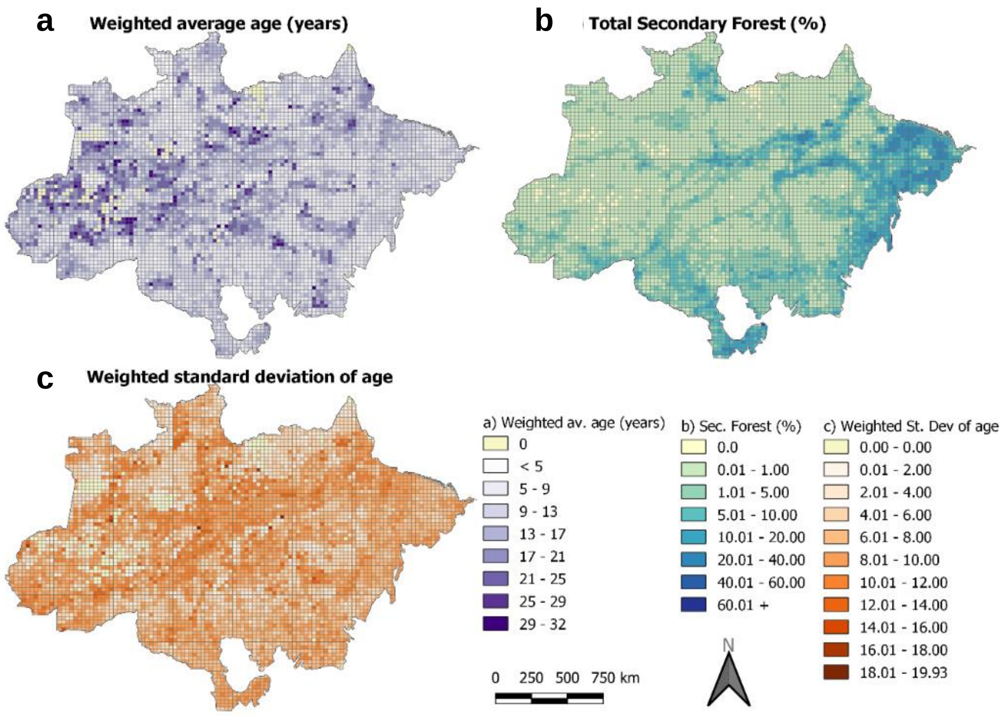

Supplementary Figure 4 | Summary maps of Amazonian Secondary Forests. Maps show (**a**) the weighted average (av.) age, (**b**) the distribution of secondary (Sec.) forests expressed as percentage of grid cell, and (**c**) weighted average standard (St.) deviation of age, in Amazonia in 2017 aggregated within 0.25° grid cells. The three legends correspond to the three subplots.

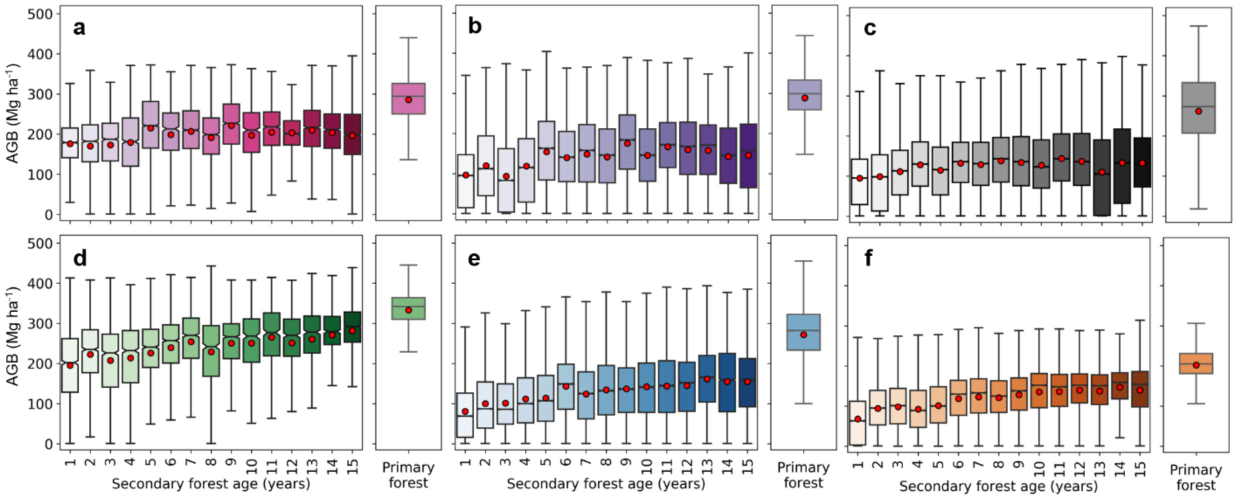

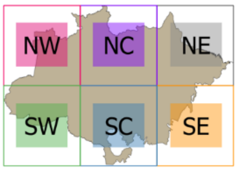

Supplementary Figure 5 | Aboveground Biomass (AGB) with secondary forest age using the Baccini et al product. The individual box plots show the spread of the AGB data observed for each secondary forest age as well as old-growth forest (Primary forest) in different regions of Amazonia for the year 2000 using Baccini et al. (2012) as input data. Subplots correspond to the regions in the bottom right inset, where (**a**) NW refers to North-West, (**b**) NC to North Central, (**c**) NE to North-East, (**d**) SW to South-West, (**e**) SC to South Central, and (**f**) SE to South-East. Box plots show the mean (red circles) and the 25th, 50th, 75th percentile of AGB for each age. The range of the data is shown, with outliers outside this region removed. The shading of the box plots simply denotes increasing age of secondary forest.

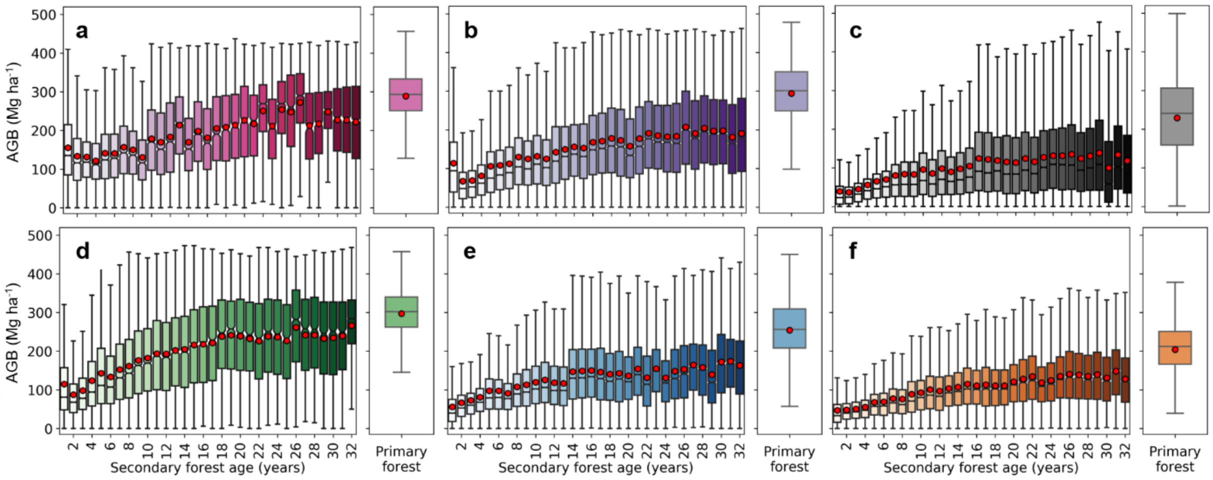

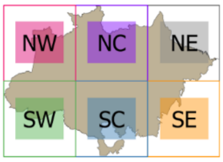

Supplementary Figure 6 | Aboveground Biomass (AGB) with secondary forest age using the ESA-CCI AGB product. As in Supplementary Figure 5 the Secondary forest growth is compared to old-growth forest (Primary forest) in different regions of Amazonia for the year 2017 using ESA 2017 biomass (Santoro and Cartus, 2019) as input data. Subplots correspond to the regions in the bottom right inset, where (**a**) NW refers to North-West, (**b**) NC to North Central, (**c**) NE to North-East, (**d**) SW to South-West, (**e**) SC to South Central, and (**f**) SE to South-East. Box plots show the mean (red circles) and the 25th, 50th, 75th percentile of AGB for each age. The range of the data is shown, with outliers outside this region removed. The shading of the box plots simply denotes increasing age of secondary forest.

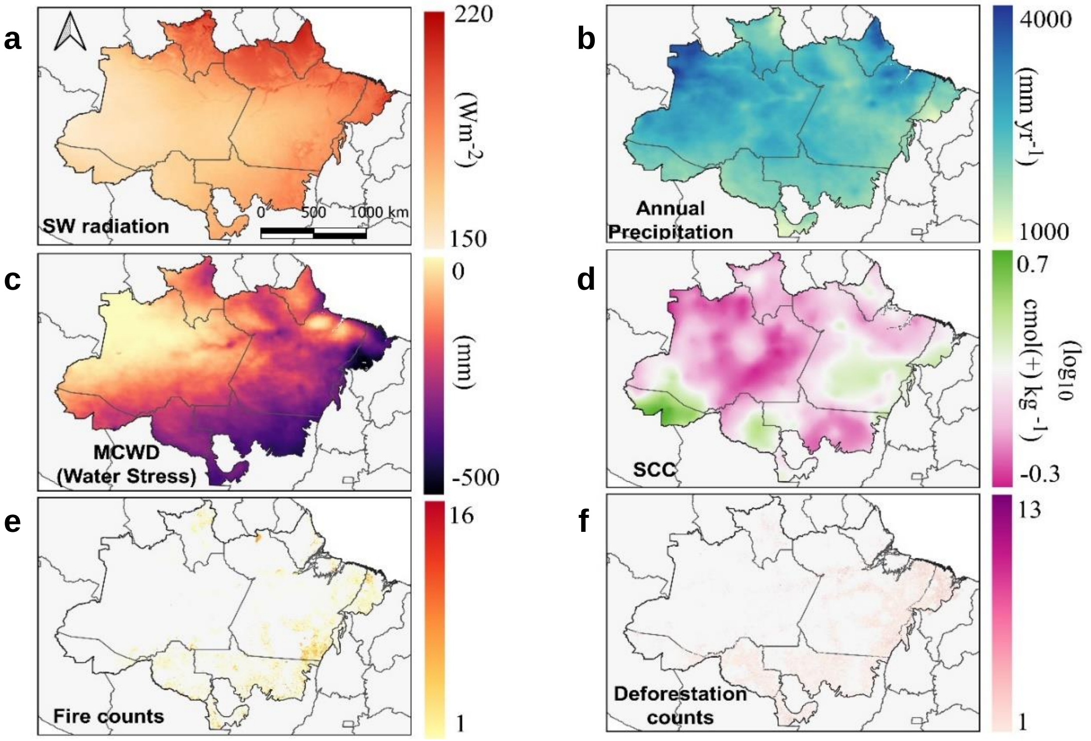

Supplementary Figure 7 | Maps of the spatial variations of the drivers used in this analysis. The drivers are arranged as they are seen in Figure 1 in the main paper. Drivers are (**a**) Annual mean downward shortwave (SW) radiation (Wm^{-2}), (**b**) Maximum Cumulative Water Deficit (MCWD; mm yr^{-1}), (**c**) Annual mean precipitation (mm yr^{-1}), (**d**) Soil Cation Concentration (SCC; log10 cmol(+) kg^{-1}), (**e**) Fire occurrences between 2001 and 2017, and (**f**) Number of deforestations prior to regrowth between 1985 and 2017, where 1 refers to areas that have only experienced the original conversion from old-growth forest to secondary forests during the period 1985 to 2017 with no subsequent deforestation events.

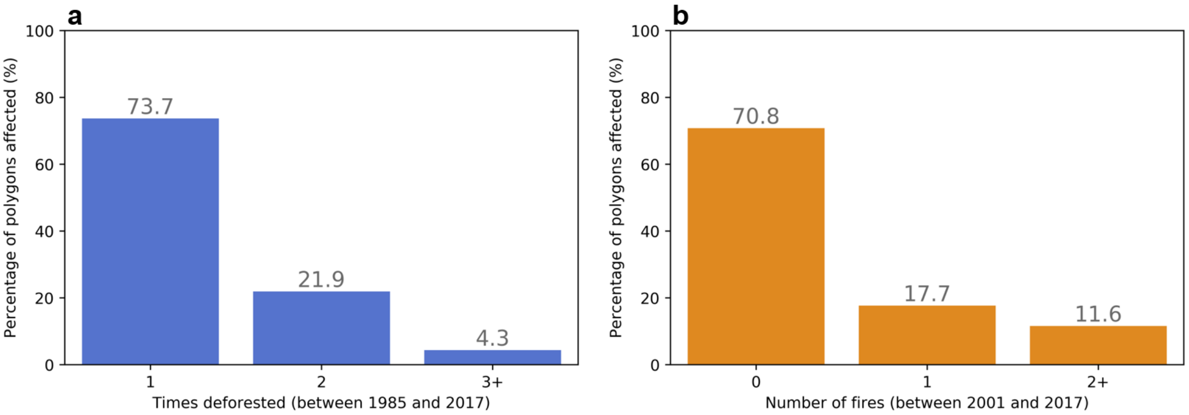

Supplementary Figure 8 | Percentage of secondary forest affected by disturbances. Percentage of secondary forests (expressed as polygons) influenced by (**a**) varying counts of repeated deforestation, and (**b**) fires (burning) across the entire Amazonian biome.

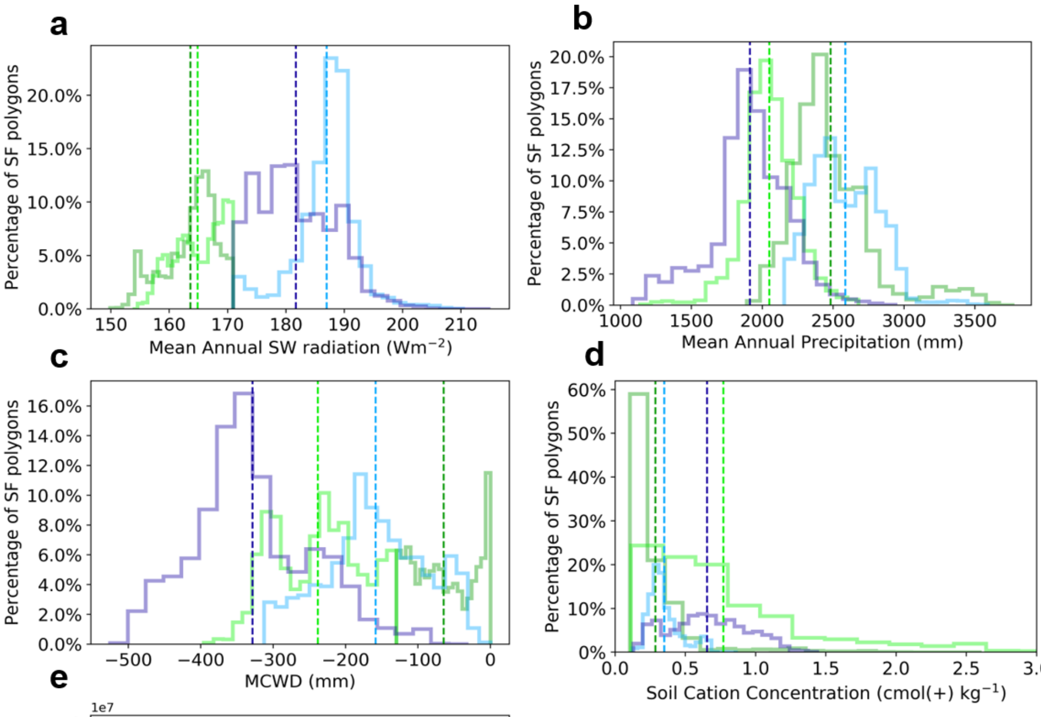

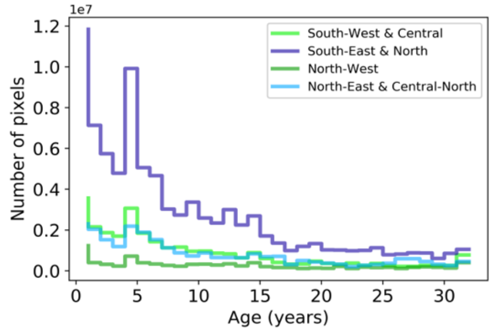

Supplementary Figure 9 | Histograms of range of the environmental and Secondary forest ages in each region. Histograms (**a-d**) refer to the four environmental variables: (**a**) Mean annual precipitation, (**b**) Maximum Cumulative Water Deficit (MCWD), (**c**) Mean annual downward shortwave radiation, and (**d**) the Soil Cation Concentration. Dotted lines denote the mean values in each location and were used to help define the qualitative interpretations of the climate ranges in each region. (**e**) The number of pixels for each age of secondary forest in each region shown in Figure 2a of the main paper.

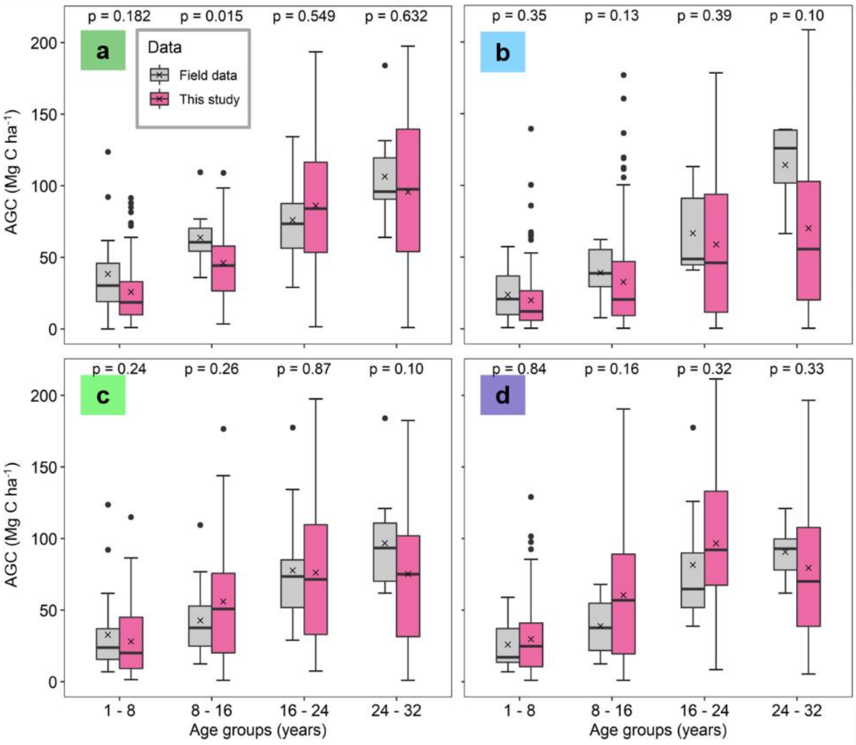

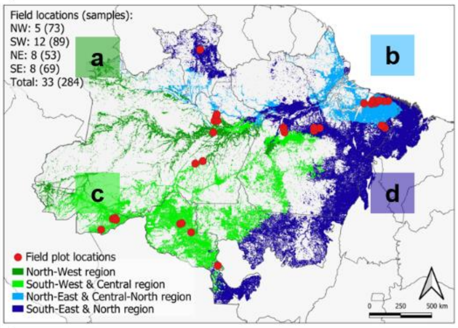

Supplementary Figure 10 | Field data and remote sensing comparison. The box plots show AGC in secondary forests obtained from field data and from the method applied in our study in the different regions of Amazonia. (**a**) North-West region, (**b**) North-East and Central-North region, (**c**) South-West and Central region, (**d**) South-East and North region. See top right inset for field plot locations, number of field plots and the secondary forest samples in each location. Plots show the interquartile range, the median (straight solid line), the mean (crosses), any outliers (black dots) as well as the p-value evaluated using the non-parametric Wilcoxon test.

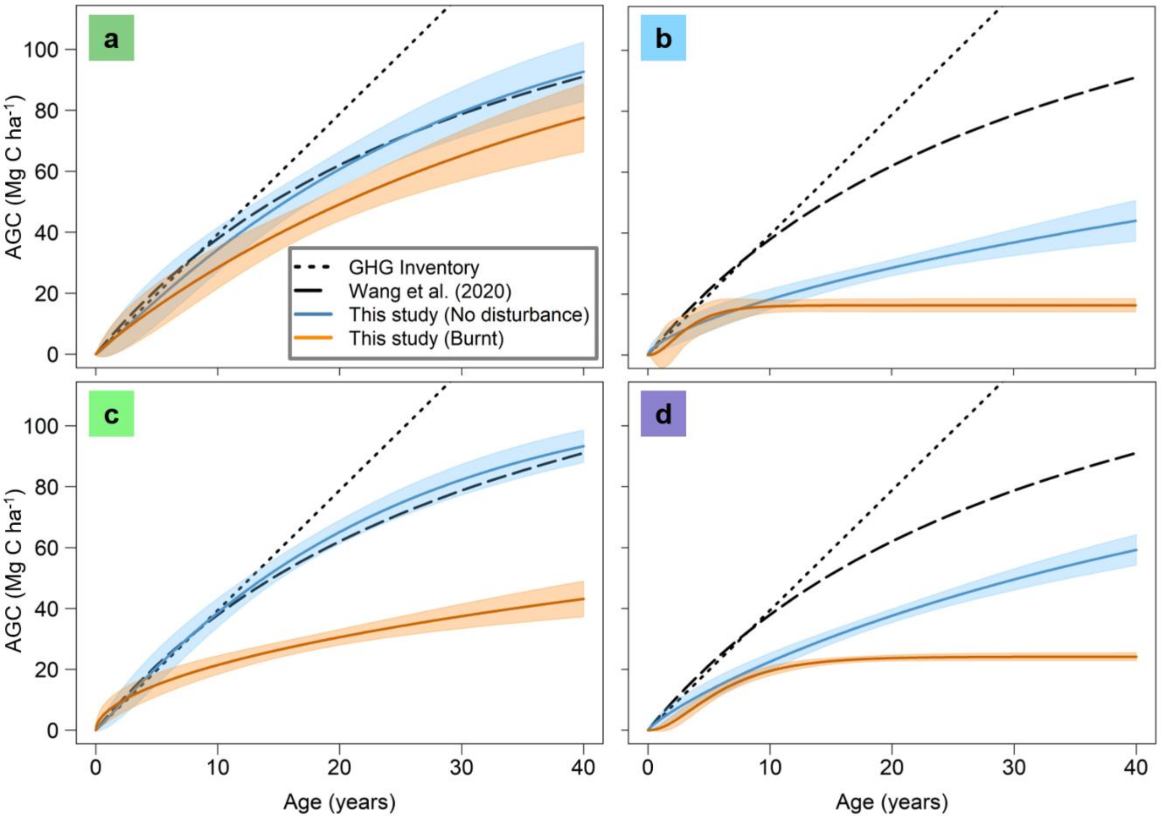

Supplementary Figure 11 | Comparison of regrowth models made in this study with models used in previous studies. The comparison was made within the four regions of the Amazon shown in Figure 2a of the main paper under different kinds of disturbance (**a – d**): (**a**) the North-West region, (**b**) the North-East and Central-North regions, (**c**) the South-West and Central region, and (**d**) the South-East and North regions. The two comparisons studies are Wang et al. (2020) and the model used in the Brazilian Greenhouse gas (GHG) inventory initially developed by Alves et al. (1997). Shading denotes the 95% confidence interval of the modelling.

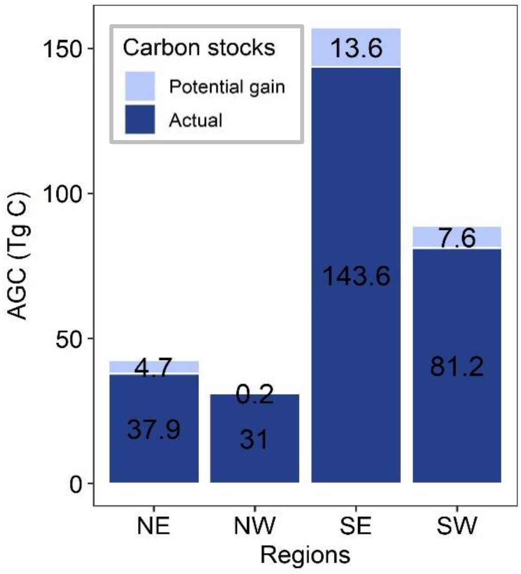

Supplementary Figure 12 | Regional analysis of estimated actual and potential carbon stocks. The carbon stock for 2017 seen in Figure 4 of the main paper broken up into the regions developed in this study. The regions have been abbreviated: North-East and Central North (NE), North-West (NW), South-East and Central (SE) and South-West and North (SW). The potential gain to the carbon stock is also shown, calculated by modelling the growth of the carbon stock through time by considering no disturbance (fire and repeated deforestation) since the initial conversion from old-growth forest to other land.

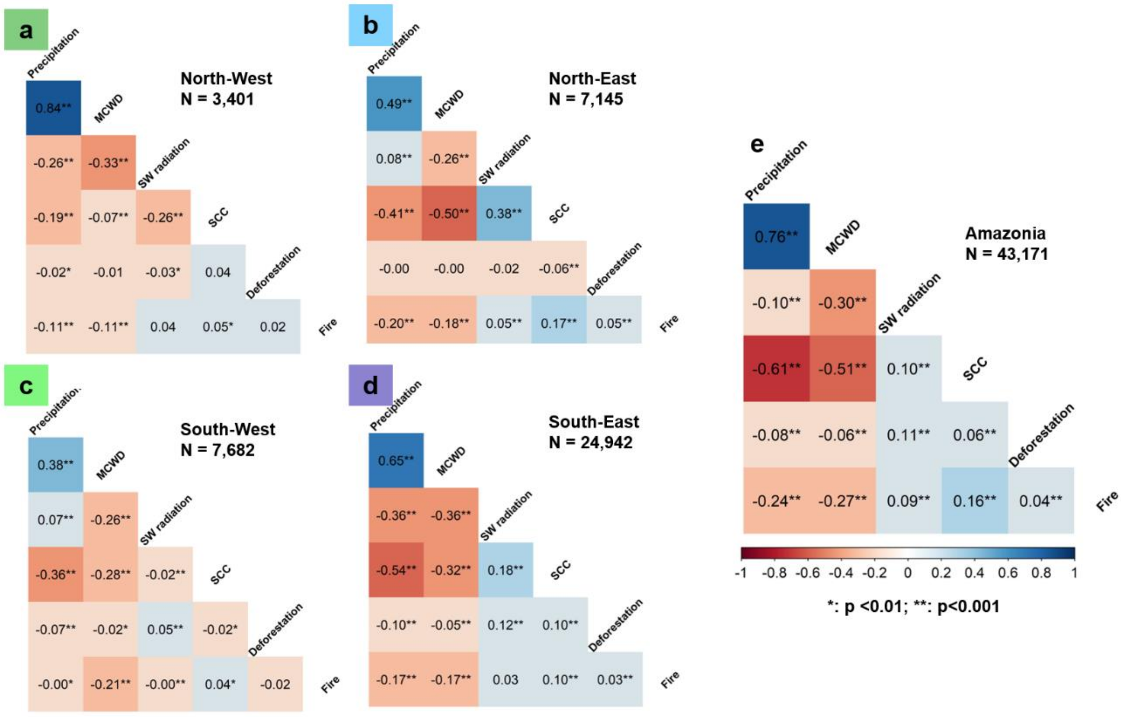

Supplementary Figure 13 | Spatial co-variation of driving variables across Amazonia. The Spearman's Rank correlation coefficient of different combinations of the driving variables in different regions across Amazonia: (**a**) the North-West region, (**b**) the North-East and Central-North regions, (**c**) the South-West and Central region, (**d**) the South-East and North regions, and (**e**) the entire biome. Stars denote statistical significance of the coefficient. The number of samples used to assess the coefficient (N) is shown in the figure. Shading denotes the sign and degree of the relationship.

Supplementary Tables

Supplementary Table 1 | Summary information of the driving variables used in this study. The products that were used to derive the variables, and the original spatial and temporal resolution of the datasets and units associated with the variable. * Units were originally in log-10 but were converted in this research.

Variable	Product used (reference)	Spatial (temporal) Resolution	Units
**Annual mean downward Shortwave Radiation**	TerraClimate - Japanese 55-year Reanalysis (JRA-55) and WorldClim v2.0 climatologies[22]	0.045° (1985 – 2017)	Wm^{-2}
**Annual mean precipitation**	Climate Hazards Group InfraRed Precipitation with Station data (CHIRPS)[23]	0.05° (1985 – 2017)	mm yr^{-1}
**Water stress**	Maximum Cumulative Water Deficit (MCWD) [23–25]	0.05° (1985 – 2017)	mm yr^{-1}
**Soil Fertility**	Soil Cation Concentration (SCC) [26]	0.1°	cation concentration cmol(+) kg^{-1} *
**Fire occurrence**	MODIS burnt area [27]	0.0045° (2001 – 2017)	Accumulated occurrence (years)
**Repeated deforestation occurrence**	MapBiomas[11] (v3.1) (this study)	0.00003° (1985 – 2017)	Accumulated occurrence (years)

Supplementary Table 2 | Statistical significance test of differences between MCWD regrowth curves. Results of the Kruskal-Wallis test, determining whether regrowth curves under different MCWD conditions were statistically significantly different. Ticks (crosses) denote that, at the 95% confidence interval, results were (not) statistically significant.

MCWD (mm yr^{-1})	> -180	-277 <= -180	-350 <= -277	< -350
> -180		✗	✗	✓
-277 <= -180	✗		✗	✓
-350 <= -277	✗	✗		✗
< -350	✓	✓	✗	

Supplementary Table 3 | Statistical significance test of differences in regrowth curves due to number of burnings. As Supplementary Table 2 but for times area experienced burning between 2001 and 2017.

Annual times burnt between 2001 and 2017	0	1	2 +
0		✓	✓
1	✓		✗
2+	✓	✗	

Supplementary Table 4 | Statistical significance test of differences between Soil Cation Concentration regrowth curves. As Supplementary Table 2 but for Soil Cation Concentration

Soil Cation Concentration (cmol(+) kg^{-1})	< 0.3	0.3 – 0.5	0.5 – 0.8	0.8 +
< 0.3		✗	✗	✗
0.3 – 0.5	✗		✗	✗
0.5 – 0.8	✗	✗		✗
0.8 +	✗	✗	✗	

Supplementary Table 5 | Statistical significance test of differences between different repeated deforestation regrowth curves. As Supplementary Table 2 but for repeated Deforestations between 1986 to 2017. Note a deforestation of 1 count implies the initial clear-cutting when the conversion from old-growth forest to other land uses took place.

Annual deforestation (counts)	1	2	3+
1		✗	✓
2	✗		✗
3+	✓	✗	

Supplementary Table 6 | Statistical significance test of differences between precipitation regrowth curves. As Supplementary Table 2 but for mean annual precipitation.

Precipitation (mm yr^{-1})	< 1920	1920 - 2210	2210 +
< 1920		✗	✓
1920 – 2210	✗		✗
2210 +	✓	✗	

Supplementary Table 7 | Statistical significance test of differences between Shortwave (SW) radiation regrowth curves. As Supplementary Table 2 but for mean annual downward shortwave radiation.

SW radiation (Wm^{-2})	< 170	170 - 180	180 - 187	187 +
<170		✗	✓	✓
170- 180	✗		✗	✓
180 -187	✓	✗		✗
187 +	✓	✓	✗	

Supplementary Table 8 | The key inputs and outputs from the regrowth models shown in Figure 1 of the main paper. Results are shown for each driver considered for analysis in this study. Corrected old-growth (primary) forest (PF) median AGC value refers to A in equation (1) of the main paper. * denotes where the model was unable to reach the old-growth forest AGC value due to the values provided by our analysis, the value in bracket therefore refers to the asymptote value reached by the model. For completeness we considered old-growth forests impacted by fire to be degraded and therefore applied the average PF value for the "No fires". [1] For units of each driver, see Supplementary Table 1. ** see Source data excel files for entire number string

Variable [1]	PF AGC (Mg C ha^{-1})	RMSE (Mg C ha^{-1})	Est. time of Asymptote (years)	Values of k and c in (1) to 3d.p**	Avg. growth rate (1 - 20 yr) (Mg C ha^{-1} yr^{-1})
**Mean MCWD (1980 – 2017)**					
**Very low water deficit (> -180)**	133	9.2	149	0.030<br>1.116	2.7 (±0.7)
**Low water deficit (-180 - -277)**	119	7.9	152	0.028<br>1.025	2.4 (±0.6)
**Moderate water deficit (-277 - --350)**	96	3.7	153	0.024<br>0.892	2.0 (±0.2)
**Very high water deficit (< -350)**	88.5	3.4	194	0.016<br>0.801	1.5 (±0.2)
**MODIS burnt area in the polygon (2001 – 2017)**					
**No Fires**	121.5	4.2	164	0.025<br>1.053	2.3 (±0.3)
**1 Fire**	121.5* (27)	(2.2)	(26)	0.216<br>2.500	1.3 (±0.4)
**2+ fires**	121.5* (20)	(4.3)	(11)	0.461<br>1.36	0.9 (±0.3)
**Soil Cation Concentration (SCC)**					
**Very low SCC (<0.3)**	124.5	5.6	149	0.029<br>1.064	2.6 (±0.4)
**Low SCC (0.3 – 0.5)**	127.5	12.3	197	0.020<br>0.950	2.1 (±0.8)
**Moderate SCC (0.5 – 0.8)**	116	5.1	218	0.016<br>0.905	1.8 (±0.3)
**High SCC (>0.8)**	150	4.0	264	0.014<br>1.023	1.8 (±0.3)
**Number of times polygon was deforested (1985 – 2017)**					
**1**	124	4.0	211	0.018<br>0.927	2.0 (±0.3)
**2**	124	4.8	316	0.009<br>0.767	1.5 (±0.3)
**3+**	124* (23)	(8.4)	(19)	0.337<br>4.461	1.2 (±7.5)
**Downward shortwave radiation (W m^{-2})**					
**Very low radiation (<170)**	120.5	7.1	99	0.051<br>1.326	3.4 (±0.6)
**Low radiation (170 – 180)**	107	4.5	153	0.025<br>0.903	2.3 (±0.3)
**Moderate radiation (180-187)**	109.5	5.6	240	0.013<br>0.803	1.6 (±0.3)
**High radiation (>187)**	139* (31)	(2.4)	(38)	0.137<br>2.210	1.3 (±0.4)
**Mean annual CHIRPS Precipitation (mm) (1985 – 2017)**					
**Low precipitation (<1920)**	96	3.0	212	0.015<br>0.780	1.6 (±0.2)
**Moderate precipitation (1920 – 2210)**	112	5.7	153	0.028<br>1.114	2.1 (±0.4)
**High precipitation (>2210)**	132	6.7	162	0.027<br>1.102	2.5 (±0.5)

Supplementary Table 9 | The key input and outputs from the regrowth models shown in Figure 3 for each region identified in Figure 2a. Corrected old-growth (primary) forest (PF) median AGB value refers to A in equation (1) of the main paper. * denotes where the model was unable to reach the old-growth forest AGB value due to the values provided by our analysis, the value outside the bracket therefore refers to the asymptote value reached by the model. For units of each driver, see Supplementary Table 1. ** see Source data excel files for entire number string

Disturbances	PF AGC (Mg C ha^{-1})	RMSE (Mg C ha^{-1})	Est. time of Asymptote (years)	Values of k and c in (1) to 3d.p**	Avg. growth rate (1 - 20 yr) (Mg C ha^{-1} yr^{-1}) (95% confidence interval)
**SW region (mean MCWD -238.4; mean annual precipitation 2049.0; mean annual SW radiation 164.9; mean SCC 0.77)**					
**No disturbance**	112.5	8.7	102	0.046<br>1.066	3.2 (±0.6)
**Fire only**	112.5	6.2	394	0.004<br>0.530	1.3 (±0.3)
**Repeated deforestation only**	112.5	12.9	182	0.0190<br>0.739	2.3 (±0.7)
**Both**	(112.5)* 22	7.8	*21	0.261<br>2.500	1.1 (±1.9)
**SE region (mean MCWD -328.5; mean annual precipitation 1913.0; mean annual SW radiation 181.7; mean SCC 0.66)**					
**No disturbance**	109.5	5.3	208	0.016<br>0.835	1.8 (±0.3)
**Fire only**	(109.5)* 24	2.1	*23	0.241<br>2.298	1.2 (±0.3)
**Repeated deforestation only**	109.5	6.2	366	0.005<br>0.555	1.3 (±0.3)
**Both**	(109.5)* 20	3.0	*25	0.195<br>2.037	1.0 (±0.3)
**NW region (mean MCWD -64.4; mean annual precipitation 2481.4; mean annual SW radiation 163.6; mean SCC 0.29)**					
**No disturbance**	122.5	13.1	123	0.0371<br>1.085	3.0 (±1.0)
**Fire only**	122.5	12.6	167	0.024<br>0.945	2.4 (±0.9)
**Repeated deforestation only**	122.5	14.5	192	0.018<br>0.749	2.4 (±0.8)
**Both**	122.5	18.9	137	0.033<br>1.124	2.7 (±1.5)
**NE region (mean MCWD -158.6; mean annual precipitation 2586.1; mean annual SW radiation 187.0; mean SCC 0.35)**					
**No disturbance**	135.5	6.4	439	0.005<br>0.672	1.3 (±0.3)
**Fire only**	(135.5)* 16	4.6	*12	0.464<br>2.480	0.8 (±0.8)
**Repeated deforestation only**	135.5	5.7	408	0.007<br>0.815	1.2 (±0.3)
**Both**	(135.5)* 11	6.2	*14	0.390<br>3.472	0.6 (±3.9)

Supplementary Table 10 | Summary information of the field study compiled and used in this study. Columns show the authors, the main study areas to which these corresponded. * denotes Doctoral of Philosophy Theses or technical reports that were not published/peer reviewed.

Field Study reference	Main study areas	Reference number
Alves et al., 1997	Cacaulândia	13
Araujo et al., 2005	Castanhal	28
Brown et al., 1992	Brasileia	29
Cassol et al., 2019	Manaus, Belterra, Santa	8
Feldpausch et al., 2004; 2005	Manaus	30,31
Fujisa et al, 1998	Theobroma	32
Gehring et al., 2005	Rio Preto, Presidente Figueiredo	33
Guimarães, 1993 *	Altamira	34
Johnson et al., 2001	Peixe-boi	35
Junqueira et al., 2010	Manicoré	36
Lima et al., 2007	Manaus	37
Lisboa, 1989	Ji-Paraná	38
Lu et al., 2004	Santo Antônio do Tauá, Altamira	39
Lucas et al., 2002a, 2002b	Manaus; Belterra	40,41
Luckman et al., 1997	Belterra	42
Mackensen et al., 2000	Belém	43
Moran et al., 2000	Ponta das Pedras	44
Pereira 1996 *	Paragominas	45
Prates-Clark 2004 *	Manaus, Belterra	46
Salimon and Brown 2000	Rio Branco	47
Salomão 1994 *	Peixe-boi	48
Santos et al., 2002; 2003	Comodoro, Mucajaí; Vila São Jorge	49,50
Silva 2007 *	Manaus	51
Silva et al., 2016	Flona Tapajos	52
Sorrensen 2000	Belterra	53
Steininger, 2000	Manaus, Maniquiri	54
Salimon 2003	Rio Branco	55
Tucker et al., 1998	Igarapé-Açu, Altamira	56
Uhl et al. 1988	Paragominas	57
Vieira et al., 2003; 2004; 2006*	São Francisco do Pará; Paragominas	58–60

Supplementary Table 11 | Statistical differences between models from this study and other studies. Values show outputs from a student T-test analysis, comparing the AGC values using models produced in this research and those used in previous research and the Brazilian Greenhouse Gas Inventory. Regional models refer to those used in Supplementary Figure 11. Statistically significant differences are where P<0.01. Colours correspond to the regions identified in this research used in other figures throughout this work.

Regional Model	Wang et al. 2020 model: P-value	Greenhouse Gas Inventory model: P-value
**SW region: No disturbance**	**P=0.74**	**P = 0.02**
**SW region: fire disturbance**	**P<0.01**	**P<0.01**
**SE region: No disturbance**	**P<0.01**	**P<0.01**
**SE region: fire disturbance**	**P<0.01**	**P<0.01**
**NW region: No disturbance**	**P=0.86**	**P<0.01**
**NW region: fire disturbance**	**P=0.04**	**P<0.01**
**NE region: No disturbance**	**P<0.01**	**P<0.01**
**NE region: fire disturbance**	**P<0.01**	**P<0.01**

Supplementary References

1. Nunes, S., Jr. Oliveira, L., Siqueira, J., Morton, D. C. & Souza, C. M. Unmasking secondary vegetation dynamics in the Brazilian Amazon. *Environ. Res. Lett.* 0–27 (2020).
2. Silva Junior, C. H. L. *et al.* Benchmark maps of 33 years of secondary forest age for Brazil. *Sci. Data* **7**, 269 (2020).
3. Almeida, C. A. de *et al.* High spatial resolution land use and land cover mapping of the Brazilian legal Amazon in 2008 using Landsat-5/TM and MODIS data. *Acta Amaz.* **46**, 291–302 (2016).
4. Almeida, C. A., Valeriano, D. M., Escada, M. I. S. & Rennó, C. D. Estimativa de área de vegetação secundária na Amazônia Legal Brasileira. *Acta Amaz.* **40**, 289–302 (2010).
5. Wang, Y. *et al.* Upturn in secondary forest clearing buffers primary forest loss in the Brazilian Amazon. *Nat. Sustain.* (2020) doi:10.1038/s41893-019-0470-4.
6. Baccini, A. *et al.* Estimated carbon dioxide emissions from tropical deforestation improved by carbon-density maps. *Nat. Clim. Chang.* **2**, 182–185 (2012).
7. Aboveground live woody biomass density | Global Forest Watch Open Data Portal. https://data.globalforestwatch.org/datasets/8f93a6f94a414f9588ce4657a39c59ff_1/data.
8. Cassol, H. L. G. *et al.* Retrieving secondary forest aboveground biomass from polarimetric ALOS-2 PALSAR-2 data in the Brazilian Amazon. *Remote Sens.* **11**, 1–31 (2019).
9. Da Silva, R. D., Galvão, L. S., Dos Santos, J. R., Camila, C. V. & De Moura, Y. M. Spectral/textural attributes from ALI/EO-1 for mapping primary and secondary tropical forests and studying the relationships with biophysical parameters. *GIScience Remote Sens.* **51**, 677–694 (2014).
10. Avitabile, V. *et al.* An integrated pan-tropical biomass map using multiple reference datasets. *Glob. Chang. Biol.* **22**, 1406–1420 (2016).
11. Mapbiomas Brasil. Project MapBiomas - Collection 3.1 of Brazilian Land Cover and Use Map Series. https://mapbiomas.org/ (2018).
12. Santoro, M. & Cartus, O. ESA Biomass Climate Change Initiative (Biomass_cci): Global datasets of forest above-ground biomass for the year 2017, v1. Centre for Environmental Data Analysis. *2019* https://catalogue.ceda.ac.uk/uuid/bedc59f37c9545c981a839eb552e4084 (2019).
13. Alves, D. S. *et al.* Biomass of primary and secondary vegetation in Rondônia, Western Brazilian Amazon. *Glob. Chang. Biol.* **3**, 451–461 (1997).
14. MCT. *Third National Communication of Brazil to the United Nations Framework Convention on Climate Change – Volume III*. vol. III (2016).
15. Quesada, C. A. *et al.* Soils of Amazonia with particular reference to the RAINFOR sites. *Biogeosciences* **8**, 1415–1440 (2011).
16. Flores, B. M., Oliveira, R. S., Rowland, L., Quesada, C. A. & Lambers, H. Editorial special issue: plant-soil interactions in the Amazon rainforest. *Plant Soil* **450**, 1–9 (2020).
17. Aalto, R. *et al.* Episodic sediment accumulation on Amazonian flood plains influenced by El Niño/Southern Oscillation. *Nature* **425**, 493–497 (2003).
18. Strobl, C., Boulesteix, A. L., Kneib, T., Augustin, T. & Zeileis, A. Conditional variable importance for random forests. *BMC Bioinformatics* **9**, 1–11 (2008).
19. Strobl, C., Boulesteix, A. L., Zeileis, A. & Hothorn, T. Bias in random forest variable importance measures: Illustrations, sources and a solution. *BMC Bioinformatics* **8**, (2007).

20. Strobl, C., Hothorn, T. & Zeileis, A. Party on! A new, conditional variable importance measure available in the party package. *R J.* 14–17 (2009).

21. Hothorn, T., Hornik, K. & Zeileis, A. Unbiased recursive partitioning: A conditional inference framework. *J. Comput. Graph. Stat.* **15**, 651–674 (2006).

22. TerraClimate. TerraClimate - Climatology Lab. http://www.climatologylab.org/terraclimate.html.

23. Funk, C. *et al.* The climate hazards infrared precipitation with stations - A new environmental record for monitoring extremes. *Sci. Data* **2**, 1–21 (2015).

24. Anderson, L. O. *et al.* Vulnerability of Amazonian forests to repeated droughts. *Philos. Trans. R. Soc. B Biol. Sci.* **373**, (2018).

25. Phillips, O. L. *et al.* Drought Sensitivity of the Amazon Rainforest. **323**, 1344–1347 (2009).

26. Zuquim, G. *et al.* Making the most of scarce data: Mapping soil gradients in data-poor areas using species occurrence records. *Methods Ecol. Evol.* **10**, 788–801 (2019).

27. Didan, K. MOD13Q1 MODIS/Terra Vegetation Indices 16-Day L3 Global 250m SIN Grid V006. NASA EOSDIS Land Processes DAAC. http://doi.org/10.5067/MODIS/MOD13Q1.006. *USGS* vol. 5 2002–2015 (2015).

28. Araujo, M. . *et al.* Padrão e processo sucessionais em Florestas Secundárias de diferentes idades na Amazônia oriental. (2005).

29. Brown, F. I., Nepstad, D. C. ., Pires, I. de O. ., Luz, L. M. & Alechandre, A. S. Carbon Storage and Land-use in Extractive Reserves , Acre , Brazil Author. *Environ. Conserv.* **19**, 307–315 (1992).

30. Feldpausch, T. R., Rondon, M. A., Fernandes, E. C. M., Riha, S. J. & Wandelli, E. Carbon and nutrient accumulation in secondary forests regenerating on pastures in central Amazonia. *Ecol. Appl.* **14**, 164–176 (2004).

31. Feldpausch, T. R., Riha, S. J., Fernandes, E. C. M. & Wandelli, E. V. Development of forest structure and leaf area in secondary forests regenerating on abandoned pastures in central Amazônia. *Earth Interact.* **9**, (2005).

32. Fujisaka, S. *et al.* The effects of forest conversion on annual crops and pastures: Estimates of carbon emissions and plant species loss in a Brazilian Amazon colony. *Agric. Ecosyst. Environ.* **69**, 17–26 (1998).

33. Gehring, C., Denich, M. & Vlek, P. L. G. Resilience of secondary forest regrowth after slash-and-burn agriculture in central Amazonia. *J. Trop. Ecol.* **21**, 519–527 (2005).

34. Guimarães, W. M. Liberação de carbono e mudanças nos estoques dos nutrientes contidos na biomassa aérea e no solo resultante de queimadas de florestas secundárias em áreas de pastagens abandonadas. (Instituto Nacional de Pesquisas da Amazônia, Manaus, 1993).

35. Johnson, C. M., Vieira, I. C. G., Zarin, D. J., Frizano, J. & Johnson, A. H. Carbon and nutrient storage in primary and secondary forests in eastern Amazônia. *For. Ecol. Manage.* **147**, 245–252 (2001).

36. Junqueira, A. B., Shepard, G. H. & Clement, C. R. Secondary forests on anthropogenic soils in Brazilian Amazonia conserve agrobiodiversity. *Biodivers. Conserv.* **19**, 1933–1961 (2010).

37. Lima, A. J. N., Teixeira, L. M., Carneiro, V. M. C., Dos Santos, J. & Higuchi, N. Análise da estrutura e do estoque de fitomassa de uma floresta secundária da região de Manaus AM, dez anos após corte raso seguido de fogo. *Acta Amaz.* **37**, 49–53 (2007).

38. Lisboa, P. L. . Estudo florístico da vegetação arbórea de uma floresta secundária em Rondônia. *Bol. do Mus. Para. Emilio Goeldi* **5**, (1989).

39. Lu, D., Mausel, P., Brondízio, E. & Moran, E. Relationships between forest stand parameters and Landsat TM spectral responses in the Brazilian Amazon Basin. *For. Ecol. Manage.* **198**, 149–167 (2004).

40. Lucas, R. M., Honzák, M., Do Amaral, I., Curran, P. J. & Foody, G. M. Forest regeneration on abandoned clearances in Central Amazonia. *Int. J. Remote Sens.* **23**, 965–988 (2002).

41. Lucas, R. M., Xiao, X., Hagen, S. & Frolking, S. Evaluating TERRA-1 MODIS data for discrimination of tropical secondary forest regeneration stages in the Brazilian Legal Amazon. *Geophys. Res. Lett.* **29**, 2–5 (2002).

42. Luckman, A., Baker, J., Kuplich, T. M., Corina da Costa, F. Y. & Alejandro, C. F. A study of the relationship between radar backscatter and regenerating tropical forest biomass for spaceborne SAR instruments. *Remote Sens. Environ.* **60**, 1–13 (1997).

43. Mackensen, J., Tillery-stevens, M., Klinge, R. & Fölster, H. Site parameters, species composition, phytomass structure and element stores of a terra-firme forest in East-Amazonia, Brazil. *Plant Ecol.* **151**, 101–119 (2000).

44. Moran, E. F. *et al.* Moran et al, 2000 - former land use effects on reg forest - BZ.pdf VN - readcube.com. *For. Ecol. Manage.* **139**, 93–108 (2000).

45. Pereira, J. L. G. Estudos de áreas de florestas em regeneração através de imagens Landsat TM. (Instituto Nacional de Pesquisas Especiais, São José dos Campos, 1996).

46. Prates-Clark, C. . Remote sensing of tropical regenerating forests in the Brazilian Amazon. (University of Aberystwyth, Wales, 2004).

47. Salimon, C. I. & Brown, I. F. Reports Secondary Forests in Western Amazonia : Significant sinks for carbon released from deforestation? *Interciencia* **25**, 198–202 (2000).

48. Salomão, R. P. Estimativas de biomassa e avaliação do estoque de carbono da vegetação de florestas primárias e secundárias de diversas idades (capoeiras) na Amazônia oriental, município de Peixe-boi, Pará. (Universidade Federal do Pará, Belém, 1994).

49. Santos, J. R. *et al.* Airborne P-band SAR applied to the aboveground biomass studies in the Brazilian tropical rainforest. *Remote Sens. Environ.* **87**, 482–493 (2003).

50. Santos, J. R., Pardi Lacruz, M. S., Araujo, L. S. & Keil, M. Savanna and tropical rainforest biomass estimation and spatialization using JERS-1 data. *Int. J. Remote Sens.* **23**, 1217–1229 (2002).

51. Silva, R. . Alometria, estoque e dinâmica da biomassa de florestas primárias e secundárias na região de Manaus(AM). (Instituto Nacional de Pesquisas da Amazônia – INPA, Manaus, 2007).

52. Silva, C. V. de J., Santos, J. R. dos, Galvão, L. S., Silva, R. D. da & Moura, Y. M. Floristic and structure of an Amazonian primary forest and a chronosequence of secondary succession. *Acta Amaz.* **46**, 133–150 (2016).

53. Sorrensen, C. L. Linking smallholder land use and fire activity: Examining biomass burning in the Brazilian Lower Amazon. *For. Ecol. Manage.* **128**, 11–25 (2000).

54. Steininger, M. K. Secondary Forest Structure and Biomass Following Short and Extended Land-Use in Central and Southern Amazonia Author (s): Marc K . Steininger Published by : Cambridge University Press Stable URL : http://www.jstor.org/stable/3068680 REFERENCES Linked re. *J. Trop. Ecol.* **16**, 689–708 (2000).

55. Salimon, C. I. Respiração do solo sob florestas e pastagens na Amazônia Sul-Ocidental , Acre.

(University of São Paulo, 2003).

56. Tucker, J. M., Brondizio, E. S. & Morán, E. F. Rates of forest regrowth in Eastern Amazônia: A comparison of Altamira and Bragantina regions, pará state, Brazil. *Interciencia* **23**, 64–73 (1998).

57. Uhl, C., Buschbacher, R. & Serrão, E. A. . Abandoned Pastures in Eastern Amazonia . I . Patterns of Plant Succession. *J. Ecol.* **76**, 663–681 (1988).

58. Vieira, I. C. G. *et al.* Classifying successional forests using Landsat spectral properties and ecological characteristics in eastern Amazônia. *Remote Sens. Environ.* **87**, 470–481 (2003).

59. Vieira, S. *et al.* Forest structure and carbon dynamics in Amazonian tropical rain forests. *Oecologia* **140**, 468–479 (2004).

60. Vieira, I. C. G. & Almeida, S. A. S. Caracterização da Cobertura Vegetal e Uso da Terra, Enfatizando os Fragmentos Florestais no Centro de Endemismo Belém. *Biota Para Fase II* (2006).
